# Success rate and ART outcome of microsurgical sperm extraction in non obstructive azoospermia: A retrospective study

**DOI:** 10.18502/ijrm.v19i9.9710

**Published:** 2021-10-10

**Authors:** Serajoddin Vahidi, Ali Zare Horoki, Mostafa Hashemi Talkhooncheh, Sara Jambarsang, Laleh Dehghan Marvast, Ali Sadeghi, Samane Eskandarian

**Affiliations:** ^1^Department of Urology, Shahid Sadoughi University of Medical Sciences, Yazd, Iran.; ^2^Shahid Sadoughi University of Medical Sciences, Yazd, Iran.; ^3^Department of Bio-Statistics and Epidemiology, Shahid Sadoughi University of Medical Sciences, Yazd, Iran.; ^4^Andrology Research Center, Yazd Reproductive Sciences Institute, Shahid Sadoughi University of Medical Sciences, Yazd, Iran.; ^5^Department of Surgical Technology, Faculty of Paramedical, Shahid Sadoughi University of Medical Sciences, Yazd, Iran.

**Keywords:** Azoospermia, Fertilization, Microdissection, Testicular.

## Abstract

**Background:**

The management of non-obstructive azoospermia (NOA) disease relies on microdissection testicular sperm extraction (micro-TESE). Few studies have assessed the role of micro-TESE in men with NOA in our country.

**Objective:**

The aim of the current study was to investigate the success rate of micro-TESE.

**Materials and Methods:**

This retrospective descriptive-analytical study was conducted on 463 men with NOA in Yazd Reproductive Sciences Institute during September 2017 through September 2019. Sperm were retrieved and frozen according to the rapid sperm freezing protocol. After preparing the oocyte of the male partner's spouse, sperms were thawed and then entered the intracytoplasmic sperm injection process. The clinical pregnancy of individuals was confirmed via ultrasound. Demographic data were extracted from medical records.

**Results:**

The success rate of micro-TESE was 38% and successful fertilization, biochemical pregnancy, clinical pregnancy, and live birth were observed in 111 (85.4%), 29 (22.3%), 29 (22.3%) and 14 (10.7%) men, respectively. A significant difference was seen between the two groups, regarding age (p = 0.01). In addition, the mean follicle-stimulating hormone in men with positive micro-TESE was significantly lower than in men with negative micro-TESE (p = 0.02).

**Conclusion:**

The success of pregnancy in couples with NOA managed via micro-TESE was significant. The study found that the success rate of micro-TESE was higher in older men and in those with lower follicle-stimulating hormone levels.

## 1. Introduction

Infertility is a reproductive health problem (1) which affects approximately 15-20% of couples (2, 3). Prevalence of primary lifetime infertility in Iran is about 25% (4). Fifty percent of couple infertility may be due to male factors (solely or in combination with female factor) (5). Male factors causes may be due to genetic and congenital (Klinefelter syndrome, AZF microdeletion, cryptorchidism, etc.) or acquired causes (surgery, medication, lifestyle, environmental pollution, etc.) (6). The most severe form of male infertility is azoospermia, which is indicated by a lack of spermatozoa in semen analyses (2). Azoospermia is seen in approximately 1% of all males and 10-15% of infertile men (7). Before the advent of intracytoplasmic sperm injection (ICSI) these men were considered to be sterile. However with the development of ICSI in 1992 they have been given a chance to own their own children (8).

Azoospermia can be categorized as obstructive (OA) or non-obstructive (NOA) form. In obstructive azoospermia, although the spermatogenesis is normal, due to genital tract obstruction, no spermatozoa can be found in the semen sample. On the other hand, in non-obstructive form, azoospermia is due to impaired spermatogenesis (9-11). NOA affects about 10% of infertile men (11) and may be due to primary (testicular) or secondary (pre-testicular) causes. NOA is found in approximately 60% of cases of azoospermia (12). Whereas in secondary form, non-surgical treatment may be a viable option, in primary one, surgical sperm retrieval is the main treatment. There are various sperm retrieval techniques with their own advantage and disadvantage (13). (E.g. testicular sperm aspiration or TESA, testicular sperm extraction or TESE, micro surgical testicular sperm extraction or micro-TESE, etc.). The ideal technique should be minimally invasive and has low risk of testicular function damage.

Micro-TESE (was first described by Schlegel) is now considered as gold standard sperm retrieval method due to its probable higher success rate and also lower testicular damage (14). It is performed via a surgical microscope to recognize dilated seminiferous tubules which may include spermatozoa. Some studies have shown that micro-TESE can improve sperm retrieval by 45-63%, compared to conventional TESE (15, 16). In a previous meta-analysis, sperm retrieval rate of micro-TESE is about 1.5 times more than conventional TESE (17).

There are several reports in the literature about the success rate of micro-TESE. Yazd Center for Infertility was the first infertility center in Iran (founded in 1992), with more than 1200 ICSI cycle per yr. The current study aimed to assess the sperm retrieval success rate of micro-TESE in this center.

## 2. Materials and Methods

This retrospective study was conducted from September 2017 to September 2019 on 463 infertile couples in whom men presented with non-obstructive azoospermia to refer to the Yazd Reproductive Sciences Institute. Azoospermia was recognized when sperm were absent in two semen samples after centrifuge and screening using an inverted microscope, according to WHO guideline 2010 (9). Information including age, smoking status, addiction history, testicular size, body mass index (BMI), and serum FSH was extracted from medical records. Inclusion criteria was male partner with small tests (testicular long axis less than 4.6 cm) and increased serum FSH (> 7.6 IU/ml) or those with normal testicular volume and serum FSH as well as negative previous testicular biopsy. Patients with endocrinopathy (hypogonadotropic hypogonadism) history of surgery (vasectomy or bilateral inguinal surgery), specific genetic anomalies (AZF a or b microdeletion, 46 XX male syndrome, etc.), were excluded from the study.

### Surgical technique

Participants underwent microscopic surgery to extract sperm. After initial preparation, in the supine position, under general or spinal anesthesia the larger testicle was delivered by transverse incision of the scrotum. According to Schlegel technique a single incision near the mid portion of testis was made to open testicular parenchyma without affecting of blood supply. Then an operating microscope (Zeiss OPMI Vario 700 Surgical Microscope) at ×20-25 magnification was focused on the seminiferous tubules and larger and whiter tubules were recognized. Small (2-10 mg) samples were obtained from these sites, taking into account how to cause the least vascular damage. They were placed in petri dishes containing human tubular fluid and the sperm were assessed by an experienced laboratory technician. If no spermatozoa were observed, the similar procedure was performed on the contralateral side. The procedure was terminated when suitable sperms was extracted or impair testis blood supply. If the sperm were suitable, rapid sperm freezing was carried out in accordance with the appropriate protocols (16).

### Sperm freezing and thawing

The retrieved sample was washed in a petri dish containing 1-2 ml Hams F10 medium supplemented with 5% serum albumin. The dishes explore at ×400 magnification under inverted microscope then cells aspirated and transferred to the sterile centrifuge tube and after adding 3 ml fresh culture medium centrifuged (300 g at 10 min). Supernatants evacuate and 0.5-1 ml culture medium added, then freezing process was performed. The sample diluted with equal volume of sperm freezing medium, which was incubated for 10 min in room temperature and then shared to the vials. The vials placed 4-5 cm above the liquid nitrogen for 1 hr and after that the vials immersed in liquid nitrogen and put in cryopreservation cane and transferred to a sperm storage bank.

The thawing process was performed according to the following process: the sample was transferred to room temperature 2-3 min and the cap was loosed then placed in a warmer at 37°C for 20-30 min; 0.5-1 ml culture medium were added to it and it was centrifuged (300 g at 5 min). after remove of supernatants fresh medium added (18).

After preparing the oocyte of the patient's spouse, the sperms were thawed and entered the ICSI process.

Positive fertilization rate is defined as transforming micro injected oocytes into two pronuclei (19). Biochemical pregnancy defined as positive serum BHCG two wk post transfer. Clinical pregnancy considered as a gestational sac diagnosed by ultrasonography (20).

Then, after the laboratory process and the formation of 4-8 cells, the fetus was transferred to the mother's uterus and after 2 wk, the mother's pregnancy was confirmed by serum beta-human chorionic gonadotropin test. The clinical pregnancy of individuals was confirmed with ultrasound.

If the micro-TESE outcome was positive men were referred for further treatment and successful fertilization, biochemical pregnancy, clinical pregnancy, and live birth were evaluated.

### Ethical considerations

Consent was obtained from all participants. The study was approved by the ethics committee of School of Medicine, Shahid Sadoughi University of Medical Sciences (Code: IR.SSU.MEDICINE.REC.1398.306).

### Statistical analysis

Data were analyzed through the Statistical Package for the Social Sciences (SPSS), version 19 software (IBM Corporation). Chi-square test, *t* test and analysis of variance were used for data analysis. A p < 0.05 was assumed to be significant.

## 3. Results

This study was conducted on 463 cases with NOA over a 2 yr period. Suitable sperm retrieval and unsuitable sperm retrieval were observed in 165 (35.6%) and 11 (2.4%) cases, respectively. No sperm retrieval was seen in 287 (62%) men. In total, out of the 463 cases, 298 participants did not continue treatment due to the failure of sperm or inadequate sperm retrieval. Moreover, 35 individuals were not referred to the infertility center. Therefore, 130 men underwent a micro-TESE-ICSI cycle. Successful fertilization, biochemical pregnancy, clinical pregnancy and live birth were seen in 111 (85.4%), 29 (22.3%), 29 (22.3%), and 14 men (10.75%), respectively.

Table I shows the frequency of underlying diseases and past procedures in the participants with positive and negative micro-TESE outcomes. The most common of these in the group with positive micro-TESE outcomes were cryptorchidism and a history of varicocelectomy; a large number of cases also had no medical history. Table II shows the findings of micro-TESE-ICSI in participants with each of the underlying diseases. As demonstrated in this table, there was no significant difference between the underlying disease groups regarding micro-TESE success rates (successful fertilization, biochemical pregnancy, clinical pregnancy and live birth) (p > 0.05). The frequency and mean of key parameters including age, testicular size, BMI, and FSH in cases with positive and negative micro-TESE outcomes are shown in Table III. A significant difference was observed between the two groups depending on age and FSH (p < 0.01). In addition, no significant difference was found between the two groups regarding smoking (p = 0.92) or addiction history (p = 0.23).

**Table 1:** The frequency of underlying diseases in positive and negative micro-TESE outcomes


**Underlying diseases**	**Positive micro-TESE outcomes (group 1)**	**Negative micro-TESE outcomes (group 2)**
**Cryptorchidism (Undescended testicle** ***)*** ****	22 (52.4)	20 (47.6)
**Klinefelter syndrome**	7 (30.4)	16 (69.6)
**Chemotherapy history**	3 (42.9)	4 (57.1)
**Varicocelectomy history**	25 (37.3)	42 (62.7)
**Varicocele history**	9 (37.5)	15 (62.5)
**Inguinal hernia surgery**	7 (35.0)	13 (65.0)
**No medical history**	103 (36.8)	177 (63.2)
Data presented as n (%), TESE: Testicular sperm extraction		

**Table 2:** The findings of micro-TESE in each of the underlying diseases


**Underlying diseases**	**Successful fertilization**	**Biochemical pregnancy**	**Clinical pregnancy**	**Live birth**
**Cryptorchidism (Undescended testicle)**	15 (78.9)	4 (21.0)	4 (21.0)	1 (5.2)
**Klinefelter syndrome**	6 (100)	3 (50.0)	3 (50.0)	0
**Chemotherapy history**	2 (100)	0	0	0
**Varicocelectomy history**	17 (89.5)	2 (10.5)	2 (10.5)	1 (5.3)
**Varicocele history**	4 (100)	2 (50.0)	2 (50.0)	2 (50.0)
**Inguinal hernia surgery**	4 (80)	1 (20.0)	1 (20.0)	0
**No medical history**	63 (82.9)	17 (22.3)	17 (22.3)	10 (13.2)
**p-value**	0.63	0.38	0.38	0.14
Data presented as n (%), Chi-square test				

**Table 3:** Frequency and mean of key parameters in men with positive and negative micro-TESE outcomes


**Parameters**	**Positive micro-TESE outcomes (group 1)**	**Negative micro-TESE outcomes (group 2)**	**p-value**
**Age**	176 (35.20 ± 6.00) (22-64)	287 (33.88 ± 5.12) (20-51)	0.01
**Testicular size**	160 (13.78 ± 6.50) (3-25)	274 (12.81 ± 6.54) (1-30)	0.13
**BMI**	36 (25.50 ± 4.81) (17.7-41.4)	61 (25.50 ± 4.21) (17.6-39.0)	0.99
**FSH**	92 (14.42 ± 13.10) (0.7-59.0)	163 (19.22 ± 16.89) (0.8-100)	0.019
Data presented as frequency, (Mean ± SD) and (minimum-maximum), *t* test, BMI: Body mass index, FSH: Follicle-stimulating hormone			

## 4. Discussion

In the current study, the sperm retrieval success rate of micro-TESE was 38% which comparable with the previous reports (22 to 77%). For instance, two similar studies on infertile men with NOA showed a success rate of 63% and 44.6%, respectively (21, 22). There is also reports about the success rate of micro-TESE in patients with Klinefelter syndrome which denote a SRR around 28% (23). Therefore, Micro-TESE could be a viable option as first line treatment in patients with non-obstructive azoospermia.

The current study also showed that male partner age may have significant effect on micro-TESE sperm retrieval success rate. Previous studies have been inconsistent in their findings regarding the effect of age on micro-TESE success (23-25) whereas, a study from Iran stated that SRR may significantly decrease in older men in comparison with their younger counterpart, another study from united states showed contrary results (23, 24).

The latter explained that this discrepancy may be due to higher prevalence of age-related secondary hypogonadism (and resultant hypo spermatogenesis) in older men in comparison with probable congenital origin of NOA in younger male participants (23). There is also reports about the effects of various factors (such as hormonal profile, previous testicular biopsy results, etc.) on sperm retrieval success rate of Micro-TESE procedure. However, most studies stated that increased age should not be an obstacle for performing micro-TESE (23, 24, 26).

In our study BMI didn't have significant affect on SRR. A study in 2015 reported that male obesity via changing hormone levels affected the composition and function of the sperm they examined (27).

Another important finding in the current study was the probable inverse relation between serum FSH and micro-TESE SRR. This is in line with another similar study which showed that higher serum FSH level may be associated with lower SRR (28). A different FSH cutoff values have been proposed for SRR in patients with NOA (29, 30, 31). However, serum FSH level should not preclude patients from micro-TESE procedure (29).

It has been reported that, the most common chromosomal anomaly in men with NOA is Kleinfelter syndrome (KS) (23, 32). The reported SRR of micro-TESE in Ks syndrome was between 21-72%. The prevalence of KS and SRR rate, in the curret study were 23 and 30.4% respectively. Therefore, it is reasonable to offer micro-TESE to all male partners with KS syndrome, despite its low success rate.

In addition, a previous study showed that the strongest predictor of the success rate of sperm retrieval was testicular histopathology (33), which we did not examine in the present study. However, performing a biopsy before surgery is not recommended. One reason is that spermatogenesis may be active even in the unsuccessful pathologies. In addition, stress before performing micro-TESE can eliminate sperm production and reduce the chances of success. Therefore, due to the potential side effects of biopsies, it is not recommended to do one before the operation (34). However, the European Association of Urology recommends that a testicular biopsy is performed at the same time as the micro-TESE procedure (26).

Our study found that living birth occurred in 11% of the NOA participants. This rate was lower than those reported in other studies (35, 36). The lower birth rate in our study may have been due to laboratory problems and fetal transmission (37).

The main limitations of the study were: the small sample size; incomplete participant's information; a lack of time to perform orchiopexy in men with undescended testis and lack of testicular biopsy for confirmed pathology.

## 5. Conclusion

The success of pregnancy in couples with NOA who were treated via micro-TESE was significant and so this may be an effective way for these couples to achieve the beautiful experience of parenthood. This study also found that the success rate of micro-TESE was higher in older men participated and in those with lower FSH. However, further studies are needed to confirm these results.

##  Conflict of Interest 

There was no conflict of interest.
